# cAMRah: a scalable and portable workflow for harmonized antimicrobial resistance gene prediction from bacterial genomes

**DOI:** 10.1093/bioadv/vbag017

**Published:** 2026-01-21

**Authors:** Daniella L Matute, Thomas H Clarke, Andrew R LaPointe, Indresh Singh, Derrick E Fouts

**Affiliations:** J. Craig Venter Institute, Rockville, MD 20850, United States; J. Craig Venter Institute, Rockville, MD 20850, United States; J. Craig Venter Institute, Rockville, MD 20850, United States; J. Craig Venter Institute, Rockville, MD 20850, United States; J. Craig Venter Institute, Rockville, MD 20850, United States

## Abstract

**Summary:**

cAMRah is a curated workflow designed to predict antimicrobial resistance (AMR) genes in microbial genomes, either in the cloud or on any personal computer running Docker containers. Numerous AMR gene-finding packages exist, each utilizing different algorithms and prediction methods. cAMRah adopts a consensus-based approach to AMR prediction, recognizing that no single tool can identify all AMR genes. It integrates and runs six AMR-finder tools and databases (with plans for future expansion), scores the AMR predictions, maps all results to CDS coordinates and harmonizes the annotation, resulting in consistent gene symbols and ontologies.

**Availability and implementation:**

Source code, demo data and detailed documentation are freely available at https://github.com/JCVenterInstitute/CAMRA.

## 1 Introduction

Antimicrobial resistance (AMR) is a global threat. A 2024 study evaluating the global AMR burden during 2021 estimated 1.14 million deaths attributable to AMR, and 4.71 million deaths associated with AMR occurrence. By 2050 they estimate 1.91 million annual deaths globally and 8.22 million associatively (GBD 2021 Antimicrobial Resistance Collaborators 2024). Antibiotic resistance in the US alone is responsible for 23 000 deaths each year with an economic impact estimated at $20 billion and $35 billion in healthcare costs and societal loss of productivity, respectively (*Antibiotic Resistance Threats in the United States* 2013).

To aid in understanding the genetic basis of resistance and global spread, high throughput genomic sequencing has significantly bolstered epidemiological investigations and surveillance efforts (Baker *et al.* 2023, Duan *et al.* 2025). Due to increased availability of high-quality bacterial genomes, many AMR gene (AMG) prediction software tools and pipelines have been developed, with over 18 that are commonly used open-sourced command line tools (Mendes *et al.* 2024). Despite all the advancements in AMG detection software, no single tool can accurately predict every AMG. Further confounding efforts to compare multiple AMG detection tools and combine multiple predictions is that each tool uses different algorithms and databases as well as various non-standardized output file formats (Kordova *et al.* 2025). Efforts have been made to “harmonize” the results of multiple AMG detection tools (Mendes *et al.* 2024), but fail to map predictions to the same genetic loci.

With all the various AMG detection tools requiring a multitude of software and library dependencies, specific hardware platforms and computing knowledge, it is challenging for biologists and healthcare professionals to run large batches of genomes. With this in mind, AMRColab was created, which is a Google-based Jupyter Notebook for running AMRFinderPlus, ResFinder, a harmonization module and geographical visualization module (Lam *et al.* 2024); however, it only runs two AMG prediction tools, suffers from the same harmonization limitations and is not portable.

Here we introduce cAMRah, a portable, reproducible workflow that runs and combines the output of six AMG detection tools, including the k-mer-based novel AMG predictor from the BV-BRC to generate a conservative and comprehensive representation of the AMR features of a bacterial genome. The aims were inspired by (Baichoo *et al.* 2018, *GLASS Whole-Genome Sequencing for Surveillance of Antimicrobial Resistance* 2020) and are as follows: (i) make a reproducible workflow, (ii) make it portable, (iii) it should scale to the capacity available to the researcher to suit heterogeneous computing environments, (iv) harmonize outputs of AMR tools at various levels to permit ease of analysis, including CARD ontology mapping, and (v) assist in the data analysis bottleneck generated by sequencing data. The workflow leverages Docker containers and WDL workflow language for platform-independent computing and a novel AMG term harmonization heuristic to map AMG predictions to specific genetic loci.

## 2 Methods

### 2.1 Workflow description language (WDL) and docker integration

Workflow description language (WDL) is a standard for describing computational workflows, allowing parallel job execution and dependency resolution by means of a Directed Acyclic Graph (DAG). Key benefits of WDL adoption are increased automation, scalability, reproducibility and open-community support. These features provide bioinformaticians reproducibility and scalability capacity as highlighted by Baker *et al.* (2023), Baykal *et al.* (2024), and Afolayan *et al.* (2021) , who emphasize the application of workflow systems to overcome research barriers.

WDL workflows function by running commands within Docker containers, which envelops all necessary software, dependencies, and resources for a specific job. Docker containers provide a consistent environment for tool execution, while supporting portability and reliability. WDL manages the location of input and output files, and the computational resources allocated for processing. WDL was chosen due to its global adoption by researchers and its native support by the Terra.Bio cloud analysis and collaboration platform.

### 2.2 Cloud enabled

Terra.Bio is a secure, cloud-native platform for biomedical research enabling data access, pipeline execution, analysis and collaboration (https://app.terra.bio/). eLwazi Open Data Science Platform has established its own instance (https://elwazi.terra.bio/) to provide African scientists access to high-throughput, reproducible analysis capabilities while maintaining strict access and authorization control, enabling cross-consortium project collaboration. By leveraging Terra’s existing infrastructure in the African research community, we aim to contribute to the Data Science building effort of the continent.

Locally-run WDLs are useful for debugging, testing, or when data privacy is of particular concern. This platform flexibility enables scalability and democratic access to the pipeline, promoting reproducibility across environments of various-sized projects and skill levels.

### 2.3 cAMRah workflow

Whole Genome sequencing (WGS) is an essential input for antimicrobial surveillance and clinical application. The CAMRA (Combating Antibiotic-Resistant Bacteria in Africa) project required an AMR workflow compatible with eLwazi’s Terra instance and scalable across various user settings. We developed cAMRah, a single-command WDL-based pipeline that generates the outputs of multiple AMR detection tools, harmonizes output file types and data structure, and consolidates ontologies to harmonize AMR gene allele naming. The workflow includes six AMR analysis tools and databases combinations, including:

1. AMRFinderPlus v3.12.8 with the AMRFinderPlus database 2024-01-31.1 (Feldgarden *et al.* 2021),
2. Resfinder v4.5.0 with the Resfinder database v2.3.1 (Bortolaia *et al.* 2020),
3. RGI v6.0.3 with the latest CARD database (Bortolaia *et al.* 2020, Alcock *et al.* 2023),
4. Abricate v1.0.1 (Seemann 2015) with the NCBI database (Feldgarden *et al.* 2019),
5. Abricate v1.0.1 (Seemann 2015) with the ARG-ANNOT database (Gupta *et al.* 2014), and
6. the BV-BRC AMR detection tool with their manually curated data collection produced from literature, data resources, direct submissions and AMR phenotypic predictions (VanOeffelen *et al.* 2021).

**Figure 1:**
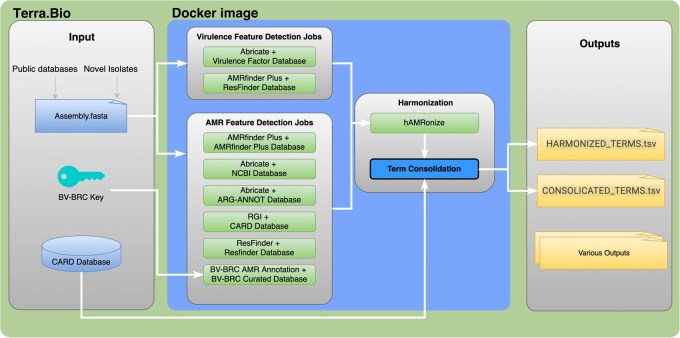
cAMRah workflow.

While there are at least 18 commonly-used AMR-finding programs that utilize different search algorithms, parameters, input and output formats, we chose four of the eight top-performing and well-supported programs that have been recently compared (Kordova *et al.* 2025). Because of its k-mer-based AMR predictive power, the BV-BRC AMR detection algorithm was also included (VanOeffelen *et al.* 2021). Using a federated workflow design, we have included the BV-BRC AMR prediction tool using the BV-BRC command-line interface tool as in Fig. 1. Users will be required to create a free account with BV-BRC to benefit from their publicly available resource.

Because each AMR annotation tool produces different output file formats, harmonization of these files is performed with *hAMRonize*, an open source tool that eases the downstream bioinformatic analysis of results (Mendes *et al.* 2024). Although hAMRonize parses 17 tools, it fails to harmonize the multitude of AMR annotations to single gene loci (Fig. 1a, available as supplementary data at *Bioinformatics Advances* online). It also does not support AMRFinderPlus version v3.12.8 or the BV-BRC AMR prediction tool; therefore, we contributed to their code by providing these advancements, and our changes were accepted by the hAMRonize Team.

### 2.4 Installation and usage instructions

Step-by-step installation and usage instructions, including the final results, can be found on our project wiki located at https://github.com/JCVenterInstitute/CAMRA/wiki/AMR-Analysis.

### 2.5 Required input and example dataset

The cAMRah workflow requires an assembly fasta file (gzip or not) and the following strings: sample_name (string), genus (string), species (string), BVBRC_username (string), and BVBRC_password (string). The required strings are entered on the submission page when running on Terra or via a JSON file when running locally using Cromwell. An example assembly fasta file and JSON file are provided as File 1, available as supplementary data at *Bioinformatics Advances* online, and on the GitHub wiki at 2.ii.a.a and 2.ii.a.b. Other optional inputs can be declared; however, they are not necessary to run the fundamental analysis but provide the opportunity to customize the workflow. If no optional inputs or parameters are provided, cAMRah will run using parameters of the AMR prediction tools as noted in Fig. 2, available as supplementary data at *Bioinformatics Advances* online.

### 2.6 AMR annotation term consolidation

Because *hAMRonize* does not perform consolidation of AMR annotation terms to specific gene loci, making the results difficult to interpret, cAMRah incorporates a term consolidation script that groups AMR hits from various tools that fall within the same loci/loci proximity (Fig. 1b, available as supplementary data at *Bioinformatics Advances* online). This was done using the following heuristic (Fig. 3, available as supplementary data at *Bioinformatics Advances* online). (i) All AMR hits are mapped to locus coordinates. (ii) The AMR hits are then perfectly matched to the CARD database based on either reference_accession, gene_symbol, gene_names or synonyms. The CARD database was chosen because it is highly curated, updated frequently and has a standardized AMR ontology to facilitate the consolidation of AMR annotation and naming conventions. Hits that did not have a CARD equivalent underwent blast-based searches. BLASTP, BLASTX, and BLASTN searches were performed against the CARD protein and nucleotide homolog and variants databases. The best quality blast alignment became the CARD match. (iii) If a query locus has only a single AMR tool hit, then its associated AMR tool annotation or CARD database match (if mapped), is reported as the consolidated term. (iv) If a query locus has multiple tool matches, then priority is given to hits with CARD exact mapping, (v) followed by AMR tool exact matches. (vi) For loci with multiple non-exact mappings, priority is given to the match with the CARD mapping with highest percent identity, (vii) followed by the AMR mappings. The pipeline returns the final output of the term consolidation WDL as (i) HARMONIZED_TERMS.tsv, which contains all the AMR hits with their CARD equivalents, and (ii) CONSOLIDATED_TERMS.tsv, which is a smaller file that contains the final decision of the identity of all the loci, ready for analysis. Users can judge the quality of the CONSOLIDATED_TERMS.tsv loci by observing the number of hits it received, the number of hits that congruently agreed on its identity and the BLAST percent identity given to the hit. The output files HARMONIZED_TERMS.tsv and CONSOLIDATED_TERMS.tsv from the cAMRah term consolidation as well as hamronize_amr_output.tsv from *hARMonize* are provided as File 2, available as supplementary data at *Bioinformatics Advances* online, and located on the GitHub wiki at 2.iii.a and 2.iii.b.

## 3 Results

cAMRah can run multiple AMG prediction tools on assembled bacterial genomes and harmonize to single genetic loci with standardized AMG ontologies assigned using a flexible, platform-independent and cloud-enabled workflow. This includes the ability to utilize the BV-BRC k-mer-based novel AMG prediction software, which can infer potential AMR phenotypes to hypothetical proteins. In addition to AMG-finding, this workflow can be adapted to predict virulence factors.

## Supplementary Material

vbag017_Supplementary_Data

## Data Availability

Source code is available on GitHub and workflow is available on Dockerstore. Github: https://github.com/JCVenterInstitute/CAMRA/tree/main Dockerstore: https://dockstore.org/workflows/github.com/JCVenterInstitute/CAMRA/CAMRA-AMR_Detection-WDL:main?tab=info
