# Supplementary material for: cAMRah: a scalable and portable workflow for harmonized antimicrobial resistance gene prediction from bacterial genomes
Source: Bioinform Adv. 2026 Jan 21;6(1):vbag017. doi: 10.1093/bioadv/vbag017 (PMC12910510; doi:10.1093/bioadv/vbag017)
Supplement: vbag017_Supplementary_Data [file vbag017_supplementary_data.zip › figureS1_final.pdf]

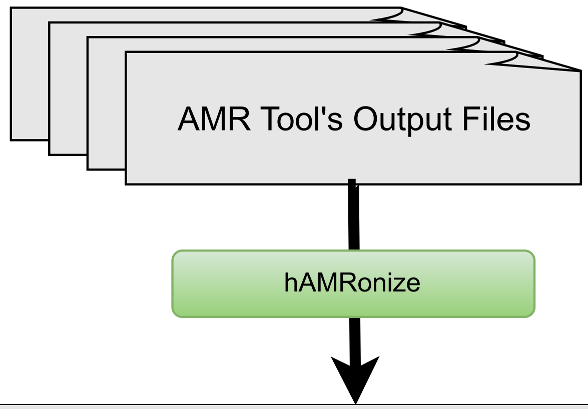

(A)

Tool/Database	gene_symbol	identity	sequence_id	gene_start	gene_stop
abricate/ arganot-db	(AGly)strB	100	contig_6	5293	6126
abricate/ ncbi-db	aph(6)-Id	100	contig_6	5293	6126
bvbrc-rast_Tk/ bvbrc-db	APH(6)-Ic/APH(6)-Id	N/A	contig_6	5293	6130
resfinder/ resfinder-db	aph(6)-Id_1	100	contig_6	5293	6126
amrfinderplus/ ncbi_reference_gene-db	aph(6)-Id	100	contig_6	5293	6126
rgi/ card-db	APH(6)-Id	99.88	contig_6	5293	6126

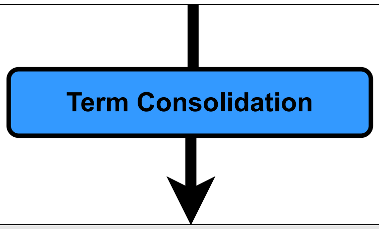

(B)

gene_name	database	ref_accession	hits	agreeing_hits	sequence_id	gene_start	gene_stop
aph(6)-Id	CARD	ARO:3002660	6	6	contig_6	6129	5293

Figure S1. Example output of harmonized AMR annotation. Depicted is harmonized output from hARMonize (**A**) compared to the annotation-harmonized output produced by the cAMRah Term Consolidation script (**B**).
