# Supplementary material for: cAMRah: a scalable and portable workflow for harmonized antimicrobial resistance gene prediction from bacterial genomes
Source: Bioinform Adv. 2026 Jan 21;6(1):vbag017. doi: 10.1093/bioadv/vbag017 (PMC12910510; doi:10.1093/bioadv/vbag017)
Supplement: vbag017_Supplementary_Data [file vbag017_supplementary_data.zip › figureS2_final.pdf]

List of Bioinformatic Tools	Software Version	Parameters	Source	Database Versions
AMRfinderPlus	3.12.8	– organism <available organism><br>– plus	docker: staphb/ncbi-amrfinderplus:3.12.8-2024-01-31.1	db 2024-01-31.1
RGI	6.0.3	pick between blast and/or diamond alignment	docker: quay.io/biocontainers/rgi:6.0.3--pyha8f3691_0	CARD v.3.3.0
Abricate	1.0.1	Default	docker: 'staphb/abricate:1.0.1-insaflu-220727'	ncbi<br><br>argannot<br>vfdb
ResFinder	ResFinder 4.5.0<br>KMA 1.4.14	– species <available species><br>--disinfectant<br>--acquired<br>--point<br>– min_cov = 0.6<br>– threshold = 0.9	docker: jcviinformaticssupport/resfinder:v4.5.0	resfinder_db=2.3.1,<br>pointfinder_db=4.1.0,<br>disinfinder_db=2.0.1
BV-BRC	CLI 1.041	– code = 11<br>– domain = “Bacteria”	docker: jcviinformaticssupport/bvbrc:5.1	BV-BRC
hAMRonize &<br>Term Consolidation	1.1.7 &<br>1.0	Default	docker: jcviinformaticssupport/hamronize:v1.1.4-build1	N/A

Figure S2. Bioinformatics tools, databases and parameters used in this study.
