# Supplementary material for: cAMRah: a scalable and portable workflow for harmonized antimicrobial resistance gene prediction from bacterial genomes
Source: Bioinform Adv. 2026 Jan 21;6(1):vbag017. doi: 10.1093/bioadv/vbag017 (PMC12910510; doi:10.1093/bioadv/vbag017)
Supplement: vbag017_Supplementary_Data [file vbag017_supplementary_data.zip › figureS3_final.pdf]

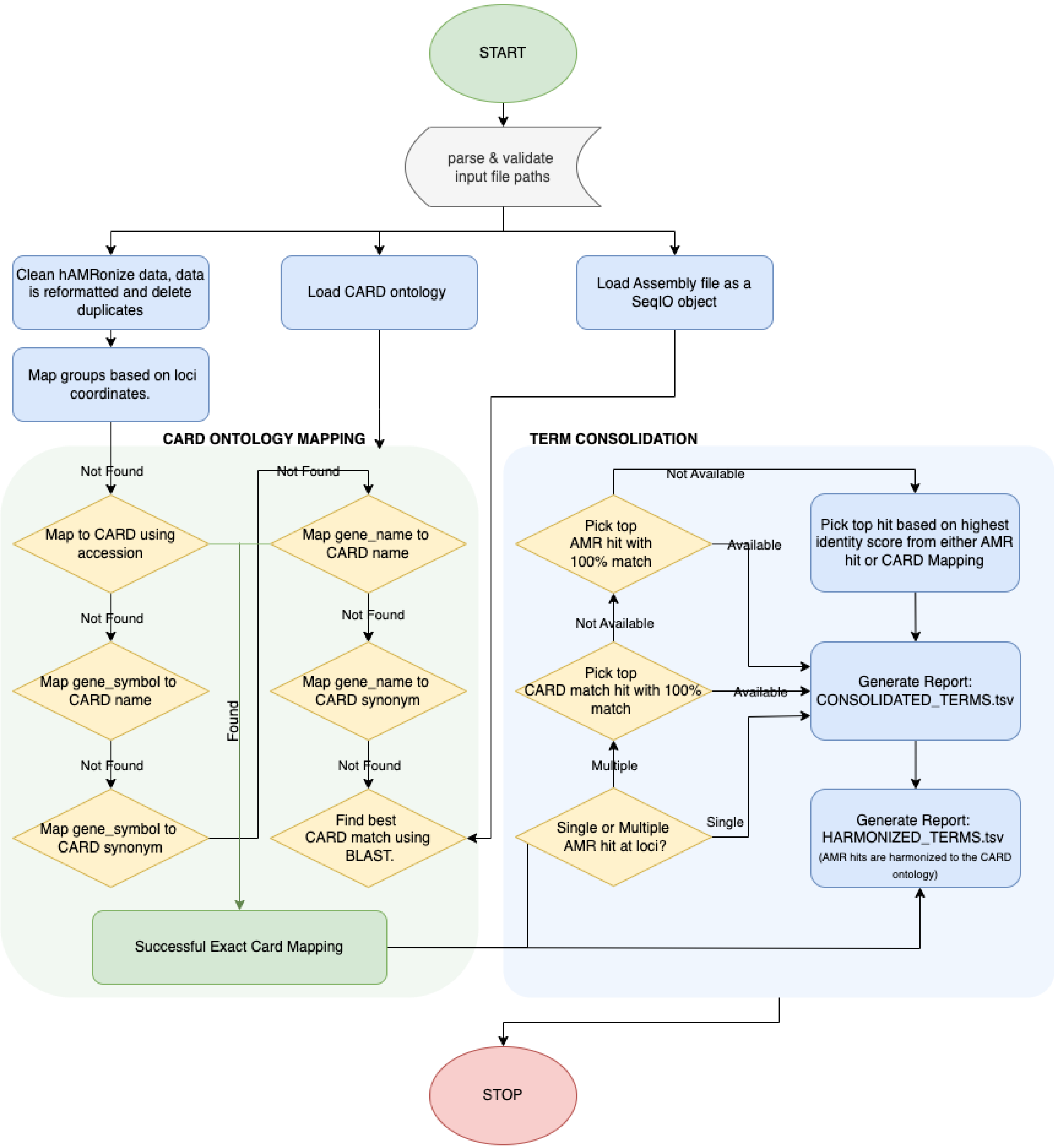

Figure S3. Flow diagram of AMR annotation harmonization by mapping and term consolidation. The diagram was rendered using draw.io.
